# Decomposition dynamics across sequential environments: subaerial and freshwater aquatic transitions

**DOI:** 10.1007/s00414-026-03887-x

**Published:** 2026-06-27

**Authors:** Alexandra Helen Lindsay, Lawrence Hill, Craig Adam Keyes

**Affiliations:** Department of Forensic Medicine and Pathology, School of Clinical Medicine, Faculty of Health Sciences, University of the Witwatersrand, Johannesburg, South Africa

**Keywords:** Decomposition, Subaerial, Aquatic, Transitional, Environment, Piglets, Scoring

## Abstract

Decomposition studies are pivotal for forensic science, offering insights into postmortem events. While decomposition dynamics in static environments are well-documented, transitions between subaerial and aquatic settings remain underexplored, particularly in diverse ecological regions. Decomposition rates were investigated in juvenile piglets subjected to subaerial-to-freshwater aquatic and freshwater aquatic-to-subaerial transitions. Twelve piglets were used in total: five in each experimental group and two controls, each exposed exclusively to either subaerial or aquatic conditions. Decomposition progression was quantified using Adjusted Total Body Scores and Total Aquatic Decomposition Scores, which were standardized for cross-environment comparison. Accumulated Degree Days were calculated to account for thermal input. The subaerial-to-aquatic transitions exhibited delayed decomposition initially, followed by rapid tissue breakdown post-submersion, often leading to near-complete skeletonization. In contrast, aquatic-to-subaerial transitions displayed rapid decomposition during submersion, followed by reduced activity and partial or complete mummification after environmental change. These shifts were reflected in scoring results but also revealed morphological changes not accounted for in current scoring systems. Transitions between environments significantly alter decomposition rates and patterns. Existing scoring systems proved limited in accounting for these shifts, particularly in cases of mummification. Findings from this study highlight the need for a novel scoring framework tailored to fluctuating environments and calls for further research into decomposition morphology specific to environmental transitions.

## Introduction

Human decomposition is a complex process that is influenced by a wide range of abiotic [1–4] and biotic factors [5–7]. In forensic science, understanding this complex process is crucial for estimating postmortem intervals (PMI), postmortem submersion intervals (PMSI), cause of death, and if the body was moved postmortem. Globally, studies on human decomposition have informed forensic practices in various climates and environmental conditions. However, decomposition rates and colonization patterns can vary significantly between different ecosystems [8], making region-specific studies essential. In many countries, the diverse environmental conditions present unique challenges and opportunities for forensic research.

Understanding the influence of dynamic environmental changes on decomposition remains a challenge in forensic research. While subaerial and aquatic decomposition processes are well documented in isolation, the effects of environmental transitions, such as in a subaerial environment and then in water or vice versa, are underexplored. Remains in natural settings can transition between subaerial and aquatic environments due to factors such as seasonal flooding [9, 10], drought, tides, and drifting of remains or deliberate disposal [11]. These transitions create unique conditions where decomposition processes could differ significantly compared to static environments.

In a static subaerial environment, the key indicators used in decomposition stage assessment include the presence and developmental stages of arthropods, algor mortis in the early stages of decomposition, livor mortis, rigor mortis, and physical changes such as skin discoloration, bloating, and tissue breakdown [12]. Subaerial decomposition is said to have a relatively faster rate of decomposition of remains compared to other environments. This can be attributed to direct sunlight, rapid insect colonization, and significant temperature fluctuation [13].

When assessing the stage of decomposition of remains found in a subaerial environment, one would use the Total Body Score (TBS) method [2]. The TBS is calculated by evaluating distinct body regions—namely the head and neck region, trunk region, and limb region—and assigning a score to each region based on observed decomposition changes. The scores are then combined to produce an overall TBS. The scoring system accounts for a variety of decomposition indicators, including color changes of the skin, presence of bloating or swelling, degree of skin slippage, development of purge fluids, and the extent of skeletonization. For the fresh stage of decomposition, there is no discoloration or apparent changes to skin across the three body regions [14]. Early decomposition is marked by the grey-green discoloration of the different body regions; however, the skin and tissue are relatively fresh [14]. Furthermore, the commencement of active decomposition is observed by the bloating of the abdomen and purging of decomposition fluids. Tissue begins to sag, and eventually bloating subsides, leading to the caving in of the abdomen. The advanced stage of decomposition is also called moist decomposition. This stage is marked by bone exposure over more than 50% of the remains: mummification is also common in this stage, as well as greasy or desiccated tissue [14]. The final stage of subaerial decomposition is when dry bone remains. However, bones may still be greasy [14, 15].

Bodies found in an aquatic environment do not exhibit the same characteristics as those found in a subaerial environment. Postmortem parameters such as algor mortis, rigor mortis, and livor mortis are altered by aquatic environments [16]. As water has a profound thermal conductivity, submersion of remains can result in significantly altered cooling patterns; thus the individual’s core temperature at the time of recovery will not aid in estimating PMI. Moreover, the floatation of the bodies creates unique scenarios and poses difficulties in establishing PMI [16]. Potential influences on decomposition have been identified to be factors such as currents and tides, dissolved oxygen levels, water acidity, depth, pressure, temperature, and debris [17, 18]. Notably, it has been found that the rate of decomposition decreases with increased depth of submergence [16–19]. When assessing the stages of decomposition of remains found in an aquatic environment, Total Aquatic Decomposition Score (TADS) [17] is used. The TADS scoring system specifically considers factors influenced by water, such as skin slippage and discoloration, presence of bloating, skeletonization, the phenomenon known as ‘washerwoman’ immersion, and adipocere formation [17]. This scoring system also works on the scale of fresh decomposition to the final stage of decomposition. In the fresh stage of decomposition, the scoring system states that there will be no apparent changes to flesh as well as no discoloration. The early stage is marked by mild bloating with yellow-green discoloration. The lips are darkened in color, and the skin starts to show signs of marbling. The active stage of decomposition has a purple to black discoloration of the skin and exposure of internal organs as well as bones. There is significant bloating as well as the onset of early stages of adipocere. The advanced stage of decomposition in an aquatic setting results in extensive bone exposure and tissue loss as well as early disarticulation of facial bones. The final stage of TADS emphasizes complete skeletonization and disarticulation, especially separation of the skull from the neck and trunk. Extensive adipocere formation is also said to be observed in the final stage.

Transitional environments in decomposition research remain poorly understood within forensic taphonomy. Previous studies have primarily examined decomposition in relation to broader seasonal or long-term environmental variation [20, 21], with limited attention given to the effects of short-term environmental transitions on a single decomposing body. As a result, the taphonomic changes associated with rapid shifts between environments, such as aquatic-to-terrestrial or terrestrial-to-aquatic exposure, remain insufficiently investigated. Given that human remains are frequently recovered from dynamic transitional contexts, understanding decomposition under these short-term fluctuating conditions is important for improving post-mortem interval (PMI) estimations and strengthening forensic reconstructions.

A study looking at the influence of rehydration on decomposing pigs in Highveld, South Africa, demonstrated that exposure to moisture led to an increase in Total Body Score (TBS) values, even after decomposition had plateaued [22]. Additionally, rehydration was associated with heightened arthropod activity, further accelerating the breakdown of soft tissue. These findings suggest that episodic moisture exposure can artificially inflate TBS values, potentially leading to an underestimation of the actual PMI. Another study conducted in the Western Cape of South Africa observed the use of an electronic sensor to quantify soft-tissue desiccation and natural mummification on a porcine model, found that previously desiccated remains were rehydrated by rainfall and saw increases in decomposition during this hydrated period [23].

Given the forensic relevance of environments and that no study, globally, has yet explicitly explored the impact of sequential environments on decomposition, further research is necessary, which could contribute to the ongoing discourse on refining PMI estimation models by accounting for unique environmental conditions. This pilot study is the first to address this limitation. The aim of the study was to describe the rate and pattern of decomposition in response to alterations in the surrounding environment of the remains from subaerial to treated freshwater aquatic conditions and vice versa and explore the effectiveness of the decomposition scoring methods (total body score and total aquatic decomposition score) on piglet bodies that decomposed in alternating environments.

## Methods

### Ethical considerations

Ethical clearance for this study was reviewed and a waiver granted by the Animal Research Ethics Committee (AREC) at the University of the Witwatersrand (Waiver No. 08-02-2024-O). This study did not involve human participants.

### Study design and setting

This longitudinal, comparative, observational study involved juvenile piglets (*Sus scrofa domesticus*) exposed to a subaerial environment and then a freshwater aquatic environment and vice versa in the Johannesburg, South Africa region. The design aimed to simulate transitional decomposition conditions relevant to forensic casework. The location for this study was a secured open lot connected to the Johannesburg Forensic Pathology Service Medico-legal Mortuary (JHB FPS).

The regional climate is classified according to the Köppen–Geiger climate classification as a subtropical highland climate, characterized by warm and wet summers and mild, dry winters [24]. The study was conducted during December 2024, a summer month associated with relatively high temperatures and afternoon showers.

A total of twelve (*n* = 12) juvenile piglet bodies (*Sus scrofa domesticus*) were included in this study. The term “piglet bodies” is used throughout to refer to the entire carcass and all associated tissues, including soft tissue and skeletal elements present during all stages of decomposition. Ten piglet bodies (1.5–6 kg) were allocated to two experimental groups: subaerial-to-aquatic group (*n* = 5) and aquatic-to-subaerial group (*n* = 5). The remaining two piglet bodies served as controls, with one that remained in the subaerial environment and the other piglet body remained in the treated freshwater aquatic environment. All piglet bodies were donated by a registered commercial pig farm and died of natural causes. A necropsy was not performed, as this would have altered decomposition processes. Natural causes of death are not expected to significantly influence decomposition rates or patterns [25].

### Environmental conditions

The subaerial environment consisted of a controlled shaded outdoor setting with direct exposure to ambient air conditions. Piglet bodies were placed on a concrete surface with naturally occurring debris, including leaves and organic material, to simulate a subaerial decomposition environment. Piglet bodies were positioned approximately one meter apart.

**Fig. 1:**
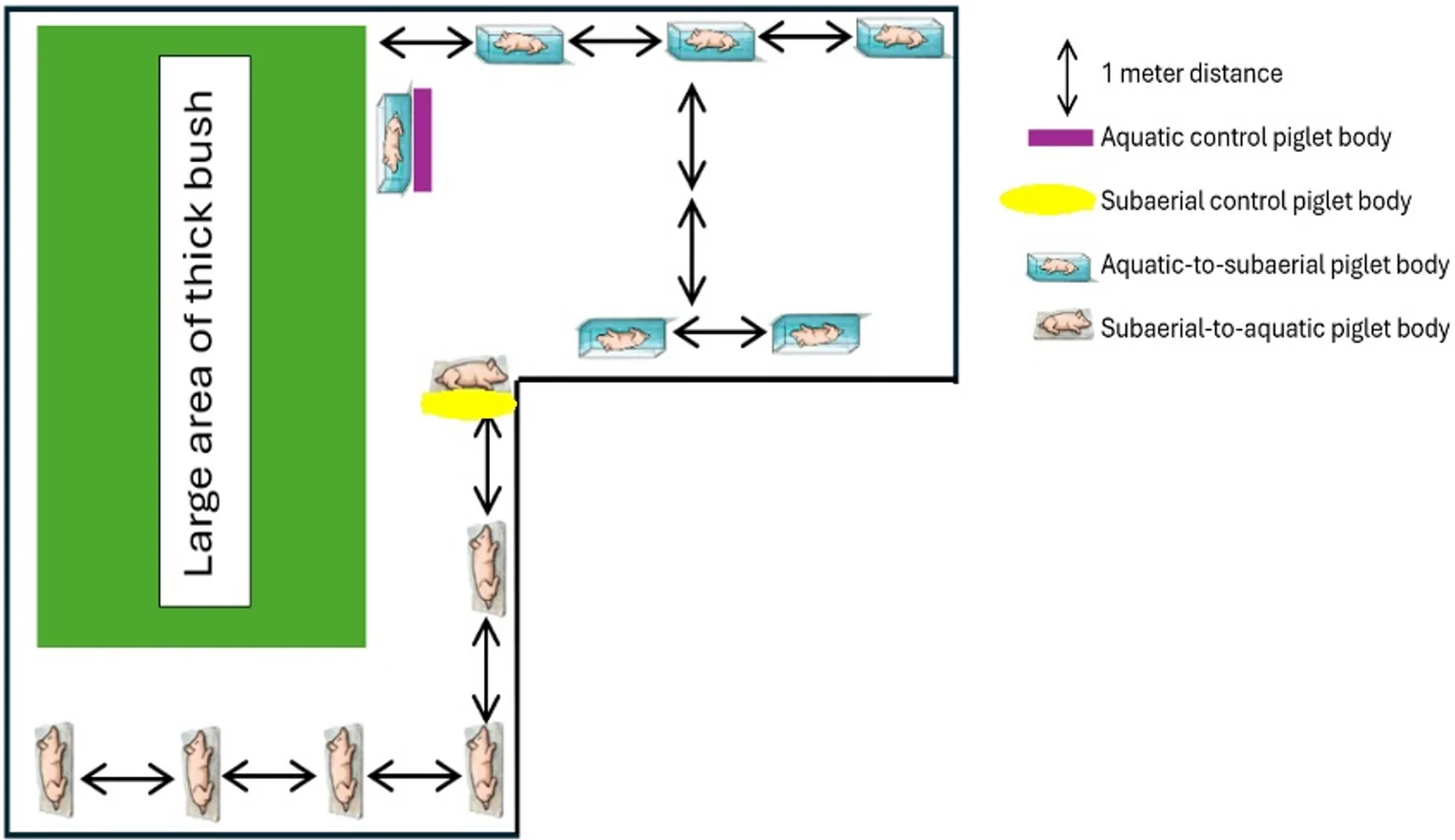
Experimental layout of piglet body placement within subaerial and treated freshwater aquatic environments. Schematic (not to scale) showing the spatial arrangement of piglet bodies in subaerial and treated freshwater aquatic environments. The subaerial-to-aquatic group, aquatic-to-subaerial group, subaerial control (yellow), and aquatic control (purple). Piglet bodies were positioned approximately 1 m apart. The aquatic environment consisted of individual uncovered containers with treated freshwater (municipal tap water), and the subaerial environment was exposed to ambient outdoor conditions on a concrete surface

The aquatic environment consisted of a controlled treated freshwater aquatic environment, created using 30-liter (54 × 36.5 × 26 cm) uncovered plastic containers filled with municipal tap water. Each piglet body was placed in an individual container one meter apart to prevent cross-contamination (Fig. 1). The water remained stagnant throughout the study period and was not replenished, as no significant loss of volume was observed due to the natural covering of the trees and periodic rainfall in the afternoon. In December 2024, Johannesburg experienced a total rainfall of approximately 107 mm to 148 mm [26]. The use of treated freshwater allowed for control of environmental variables such as water flow and chemical composition.

### Experimental procedure and environmental transitions

All twelve piglet bodies were deployed simultaneously into their allocated environments. Piglet bodies in the subaerial-to-aquatic group were initially placed in the subaerial environment for seven days, after which they were transferred to a treated freshwater aquatic environment for a further seven days. This transition involved placing the piglet bodies into new, uncontaminated 30 L plastic containers filled with municipal tap water. All associated arthropods and attached vegetation were retained during transition. Piglet bodies in the aquatic-to-subaerial group underwent the reverse sequence, remaining in the treated freshwater aquatic environment for the first seven days before being removed and placed in the subaerial environment for the remaining seven days. This transition involved removing the piglet bodies from the water-filled containers and positioning them on a concrete surface within the subaerial environment with all associated arthropods retained during the transition. The water from the containers was disposed of in accordance with biohazard safety protocols. The seven-day interval prior to environmental transition was selected to prevent piglet bodies from reaching advanced decomposition stages before the transition occurred. This timeframe was informed by decomposition rates observed in previous studies conducted at the same site [27].

Minimal disturbance to the remains was maintained throughout the study to preserve the integrity of the decomposition process. In the subaerial environment, piglet bodies were not moved once placed, except for minor adjustments necessary during scoring. In the aquatic environment, piglet bodies were only lifted while still submerged for scoring purposes (scoring of piglet bodies was done once daily for the 14-day study period). These measures were implemented to reduce external influences on decomposition patterns, thereby ensuring consistency and reliability in the observational data.

### Decomposition scoring

The piglet body’s decomposition was scored daily on-site using an adjusted total body score (ATBS) [28] developed for pig TBS scoring [2] for the subaerial environment and Total Aquatic Decomposition Score (TADS) for the aquatic environment [17]. This system was developed on human remains, introducing a limitation to the study. However, as no TADS system for pig bodies has been published to date, the human remains TADS was used with slight modification to the terminology of appendages. All scores (ATBS and TADS) were converted into a standardized TBS (STBS) (out of a total of 35) to allow for direct comparisons between the groups (subaerial-to-aquatic group and aquatic-to-subaerial group) throughout the decomposition period and environmental changes. ATBS is already out of a total score of 35, so those were not changed, but the scores for TADS are out of a total score of 24, so they were converted into an STBS out of 35 using the following equation: $\:STBS=\:\left(\frac{x}{24}\right)\times\:35$.

### Temperature recording and accumulated degree days

The ambient temperature of the subaerial and aquatic environments in each container was recorded daily at the time of each documentation session. Temperature was recorded using iButton data loggers (Maxim iButton DS1922L) in latex gloves for protection; these were secured on site and set to take temperature measurements hourly. These data were used to calculate accumulated degree days (ADD) (calculated as the sum of daily average temperatures), which was used in correlation with STBS to plot decomposition rates. ADD was calculated using the following formula: $\:ADD={\sum\left(\frac{{max\:temperature}\;+\;{min}\;temperature}{2}\right)}$. STBS was then plotted against logADD for each pig to show the change in decomposition rate during the experimental period. The logarithmically transformed ADD was used to obtain a linear association between STBS and ADD.

### Morphological assessment

To describe the pattern of decomposition in the two experimental groups, gross morphological changes of the remains were described and categorized into decomposition stages and compared between the two experimental groups. These changes were recorded and classified according to the following accepted decomposition stages: fresh, bloat, active decay, advanced decay, and skeletal remains; identified based on key visual indicators, including tissue discoloration, bloating, maggot activity, soft tissue breakdown, and skeletal exposure of the piglet bodies. These stages typically include (i) early decomposition, (ii) bloated, (iii) active decay, (iv) advanced decay, and (v) dry remains as guided by published literature [29, 30].

## Results

Decomposition progression varied notably between the subaerial-to-aquatic and aquatic-to-subaerial groups. Key findings included accelerated skeletonization in the rehydrated remains of the subaerial-to-aquatic group and the unexpected mummification of the post-submersion aquatic-to-subaerial group. The results for this study are presented in two parts: decomposition body score analysis and morphological changes.

### Decomposition score analysis

#### Days 1–7 (pre-transition phase)

During the initial seven days, piglet bodies decomposed according to the expected patterns for their respective environments (Fig. 1).

**Fig. 2:**
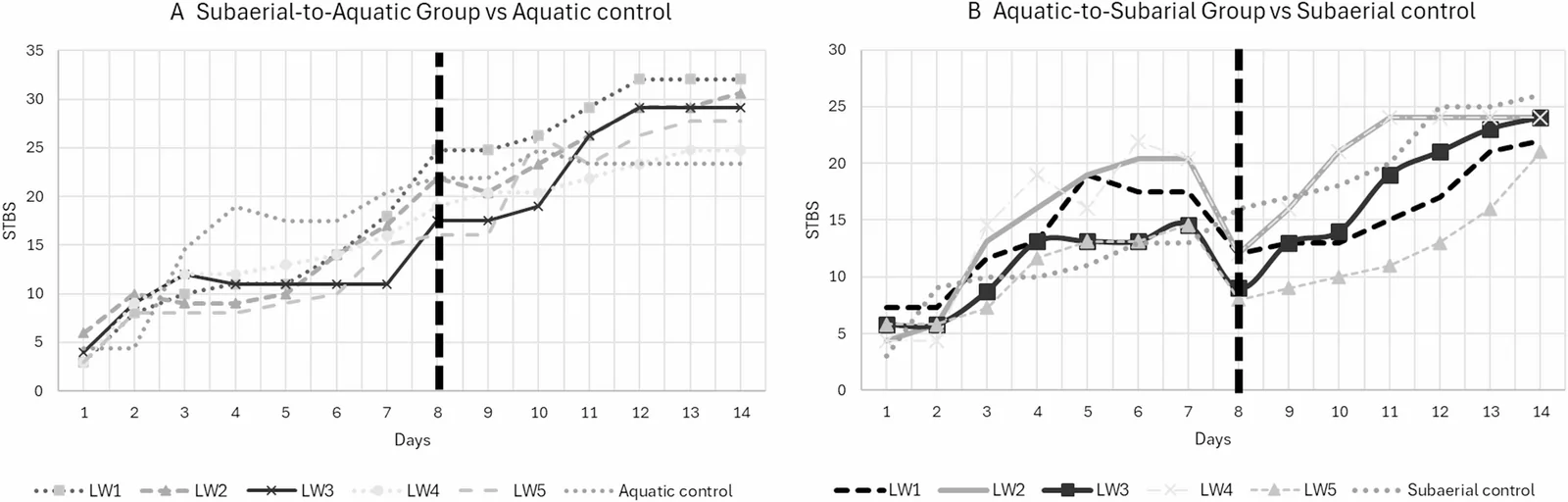
Standardized total body score (STBS) progression over time. STBS values for piglet bodies in the (**A**) subaerial-to-aquatic piglet bodies and (**B**) aquatic-to-subaerial piglet bodies across the study period. STBS was derived by standardizing adjusted total body score (ATBS) and total aquatic decomposition score (TADS) to a common scale (maximum score = 35). The environmental transition occurred at Day 7. The apparent decrease in STBS in Fig. 2B reflects the change from TADS to ATBS scoring rather than a reversal in decomposition

Piglet bodies in the subaerial environment (subaerial-to-aquatic) exhibited a steady increase in standardized total body score (STBS) values over time (Fig. 2A), reflecting progressive decomposition. Morphologically, these piglet bodies showed early drying of the epidermis, onset of bloating, purging of decomposition fluids, and increasing insect activity, consistent with established terrestrial decomposition patterns.

In contrast, piglet bodies in the treated freshwater aquatic environment (aquatic-to-subaerial) displayed slower initial increases in STBS values (Fig. 2B). Morphological changes included early and pronounced bloating, skin slippage, and discoloration, particularly in exposed areas. Insect activity was present but generally restricted to regions at or above the waterline.

By Day 7, the mean STBS values for the subaerial-to-aquatic and aquatic-to-subaerial piglet bodies were comparable (17.4 and 17.5, respectively), indicating similar overall levels of decomposition despite differing environmental conditions (Fig. 2).

### Days 8–14 (post-transition phase)

Following the environmental transition on Day 7, decomposition trajectories diverged substantially between the two experimental groups.

Piglet bodies in the subaerial-to-aquatic group, after transitioning from the terrestrial to the aquatic environment, exhibited a rapid increase in STBS values between Days 8 and 14 (Fig. 2A). By Day 14, STBS values ranged from 27.71 to 32.08, exceeding those observed in the terrestrial control piglet body (final STBS = 26.00). This acceleration in decomposition corresponded with visible rehydration of previously desiccated tissues, followed by rapid tissue sloughing and extensive skeletal exposure.

In contrast, piglet bodies in the aquatic-to-subaerial group demonstrated a decrease in STBS values immediately following the transition to the terrestrial environment (Fig. 2B). This decrease reflects a methodological shift from TADS to ATBS, rather than a true reversal in decomposition. Thereafter, STBS values increased more gradually, with final values ranging between 19.5 and 25.5 by Day 14.

Morphologically, piglet bodies in this group underwent rapid desiccation following removal from the aquatic environment. Previously hydrated tissues became dry, darkened, and leathery in appearance, consistent with mummification. This process appeared to slow further visible soft tissue decomposition despite ongoing insect activity.

### Decomposition score analysis

**Fig. 3:**
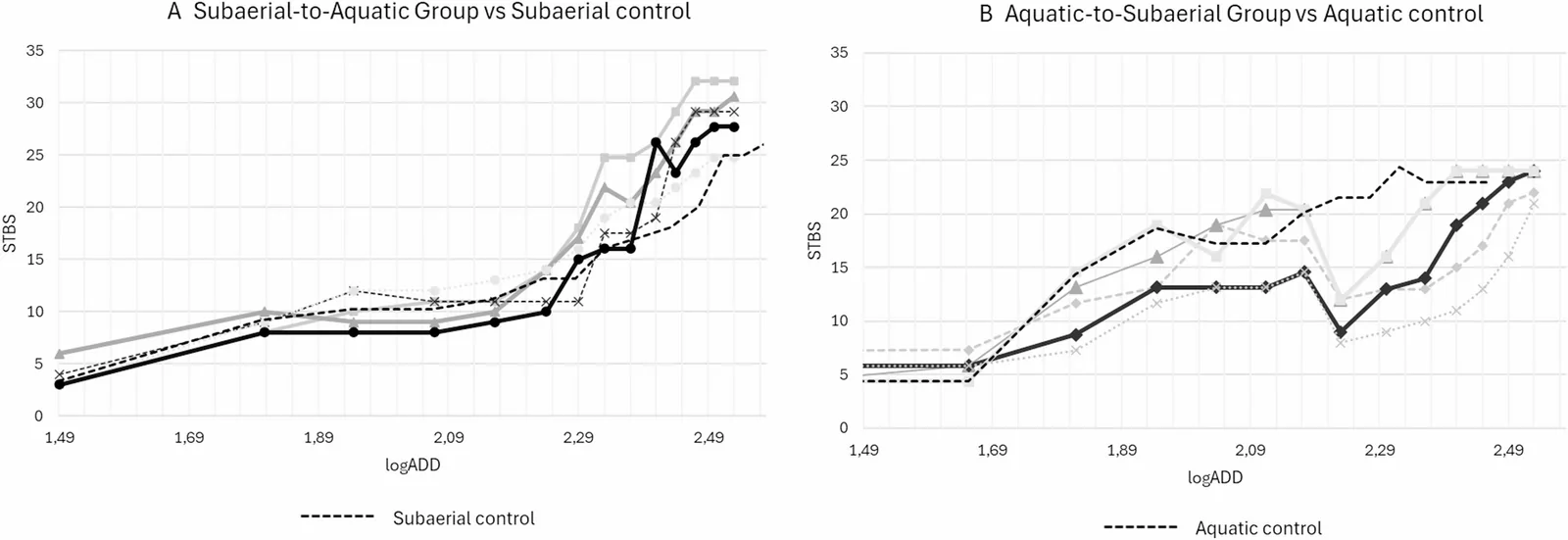
Relationship between standardized total body score (STBS) and accumulated degree days (ADD). Plots of STBS against logarithmically transformed accumulated degree days (logADD) for piglet bodies in the (**A**) subaerial-to-aquatic group and (**B**) aquatic-to-subaerial group. STBS represents a standardized decomposition score derived from adjusted total body score (ATBS) and total aquatic decomposition score (TADS). Coefficients of determination (R²) indicate the strength of the relationship between thermal input and decomposition progression

Analysis of STBS values in relation to ADD demonstrated differing decomposition patterns between the two experimental groups (Fig. 3). Piglet bodies in the subaerial-to-aquatic group showed a strong positive relationship between STBS and logADD, with individual R² values ranging from 0.70 to 0.93 (Fig. 3A). This indicates a consistent relationship between thermal input and decomposition progression in this group. In contrast, the aquatic-to-subaerial group exhibited weaker correlations between STBS and logADD (R² = 0.48–0.79), reflecting greater variability in decomposition progression following environmental transition (Fig. 3B).

The aquatic control piglet body demonstrated a steady increase in STBS, while the subaerial control decomposed more gradually, providing reference patterns for static environmental conditions.

A Friedman test revealed statistically significant differences in STBS values across time points (*p* ≤ 0.001). Post hoc Wilcoxon signed-rank tests also indicated significant differences (*p* ≤ 0.001), particularly around the transition period (Day 7–8), confirming that environmental changes had a measurable impact on decomposition progression.

### Morphological changes

At the start of the study, remains in the subaerial and aquatic environments showed the expected, well-documented morphological differences characteristic of each setting. The piglets initially placed in a subaerial environment rapidly developed drying and tightening of the epidermis, early bloating followed by purging decomposition fluids, extensive maggot-related tissue loss, and progressive desiccation of the face and extremities, consistent with commonly reported terrestrial decomposition patterns under warm conditions [2, 14]. Piglets initially submerged in a treated freshwater aquatic environment bloated early and, exhibited widespread skin slippage, and high levels of insect activity including larval feeding in accessible surface such as the face, limbs, and areas exposed above or near the waterline. However, once the experimental groups transitioned between environments, their morphological trajectories changed noticeably. Piglets that transitioned from the subaerial environment to the aquatic (subaerial-to-aquatic group) experienced rapid rehydration of previously dried tissue, resulting in accelerated tissue sloughing and subsequent skeletonization. Conversely, the piglets transitioned from an aquatic environment to a subaerial (aquatic-to-subaerial group) began to dry almost immediately; the previously hydrated, softened tissues tightened and darkened, forming a leathery, mummified exterior that slowed further soft tissue decomposition despite the presence of insects. These environmental shifts produced distinct morphological patterns not observed in static environments and demonstrate how decomposition can deviate substantially from predictable trajectories once remains are exposed to more than one environment.

## Discussion

The findings of this study provide novel insights into decomposition processes under transitional environmental conditions, highlighting the forensic relevance of rehydration-induced accelerated decomposition and mummification following aquatic exposure. These findings challenge the previous assumptions of decomposition rates in aquatic and subaerial settings and underscore the need to revise decomposition scoring tools for transitional environments.

### Decomposition body score: trends and effectiveness

Previous studies have found that decomposition generally progresses more slowly in treated freshwater aquatic environment compared to land [31–35]. However, while submerged, aquatic-to-subaerial group piglet bodies exhibited higher STBS values than subaerial-to-aquatic group piglet bodies in the subaerial environment during the initial environment period. These higher STBS values could be a result of the standardizing ATBS and TADS to a shared total using a mathematical conversion, which may unintentionally inflate the score values. This highlights the need for an aquatic scoring system that is numerically aligned with existing subaerial frameworks.

After the transition, subaerial-to-aquatic group piglet bodies in the treated freshwater aquatic environment decomposed more rapidly than aquatic-to-subaerial group piglet bodies in the subaerial environment, further reiterating that decomposition progresses faster in the treated freshwater aquatic environment than in the subaerial environment. A study done on the rehydration of porcine piglet bodies saw similar results and concluded that rehydration influenced the progression of decomposition and subsequent rate, with rehydration increasing decomposition score values [19]. This could be a result of the integrity of the skin being compromised and thus water promotes the disarticulation of the piglet bodies as well as serves to macerate the piglet bodies.

The subaerial-to-aquatic group showed a strong correlation (average R^2^ value = 0.8009) between STBS and logADD, suggesting that piglet bodies initially decomposing in the subaerial environment and later submerged can still be effectively scored using adjusted total body score (ATBS) [2, 28] and total aquatic decomposition score (TADS) [23]. This implies that PMI estimation may still be feasible in such cases. In contrast, the aquatic-to-subaerial group demonstrated poor reliability between STBS and logADD, indicating that piglet bodies initially decomposed in the treated freshwater aquatic environment and later found on land may not be accurately scored with existing systems. This suggests PMI estimation in such cases would likely be unreliable. Future research should explore how environmental transitions, particularly aquatic-to-subaerial shifts, influence decomposition dynamics and PMI accuracy.

The forensic implications of these findings extend beyond PMI estimation. Aquatic remains often present substantial challenges to forensic investigation due to advanced decomposition and prolonged submersion, which may hinder identification and reconstruction of events surrounding death [36]. Transitional decomposition pathways may further complicate these interpretations if the environmental history of the remains is not accounted for in the postmortem reconstruction.

The results of this study demonstrated that while STBS provided a means of comparing decomposition progression across different environments, the underlying scoring systems (ATBS and TADS) varied in their effectiveness. The initial similarity in STBS between the subaerial-to-aquatic group and the aquatic-to-subaerial group on Day 7 suggests that both groups experienced comparable levels of decomposition prior to the environmental switch. However, following the transition, a clear divergence in decomposition trajectories was observed. The subaerial-to-aquatic piglet bodies, transitioning from the subaerial into the treated freshwater aquatic environment, exhibited a rapid increase in STBS, whereas aquatic-to-subaerial group, transitioning from the treated freshwater aquatic environment to land, displayed a substantial decline in STBS. This decline is most likely attributed to the change in the scoring system. The aquatic-to-subaerial piglet bodies were initially scored using TADS.

Subaerial-based scoring systems, such as ATBS, do not account for the unique morphological decomposition patterns associated with aquatic environments. Aquatic decomposition is influenced by factors that can result in distinct soft tissue preservation and loss patterns that differ significantly from subaerial decomposition. Consequently, when piglet bodies transition from an aquatic to a subaerial setting, pre-existing decomposition features may not align with the criteria used in traditional subaerial scoring systems, leading to inaccurate assessments of decomposition progression.

Mummification in the aquatic-to-subaerial group may also have affected the score interpretation. Once removed from the treated freshwater aquatic environment, the hydrated tissues dried rapidly, forming a leathery exterior that slowed the visible soft-tissue breakdown. As ATBS depends on progressive tissue loss, this desiccation produced lower-than-expected scores while simultaneously giving the piglet bodies an advanced decomposition appearance. As a result, mummified piglet bodies that were previously aquatic may falsely suggest a much longer PMI if their transitional history is not known.

### Morphological changes

By the conclusion of the study, most subaerial-to-aquatic piglet bodies had reached partial to near-complete skeletonization with rapid decomposition. This is an intriguing finding, as prior studies suggest that piglet bodies that decompose primarily in aquatic environments typically do not undergo complete skeletonization [35]. For instance, in a study done in Sweden on outdoor human decomposition, of the 51 aquatic bodies included in the study, only one reached complete skeletonization [37].

The near-complete skeletonization of subaerial-to-aquatic piglet bodies could be attributed to the piglet bodies, which initially had desiccated tissue due to subaerial exposure, being rehydrated once placed into an aquatic environment. This phenomenon has been observed in a South African setting [13, 16]. International studies have reported that presence of moisture can alter decomposition trajectories by reactivating microbial activity and facilitating further soft-tissue breakdown [38, 39]. However, complete or near-complete skeletonization remains uncommon in aquatic environments, making the extent of skeletal exposure observed in the present study particularly noteworthy. The findings therefore suggest that prior subaerial exposure followed by rehydration may represent a unique decomposition pathway that differs from decomposition occurring exclusively in exclusively aquatic or terrestrial environments.

This has important forensic implications as it demonstrates that piglet bodies initially deposited in a subaerial environment may undergo accelerated soft-tissue loss once submerged in water, producing a morphological outcome that resembles long-term aquatic decomposition even when the actual time in water is short. This may lead to overestimation of the duration of submersion or misinterpretation of the body’s movement history if transitional decomposition pathways are not considered.

The aquatic-to-subaerial piglet bodies underwent mummification after transitioning to a subaerial environment. This is particularly significant as a study done on rehydrated porcine remains in Western Cape, South Africa, saw that the porcine remains that were exposed to rainfall did not later desiccate [16]. It is worth noting that rainfall occurs predominantly in the winter months in the Western Cape, whereas the present study was conducted in summer. Temperature, a key driver of decomposition, may explain this discrepancy. Furthermore, mummification requires a lack of moisture to occur [40–43], which preserves soft tissues by preventing microbial activity and decay. The findings of previously wet remains mummifying are unusual, as mummification is generally not expected to occur in tissues that have been saturated with moisture. Instead, one would anticipate continued decomposition, which is common in moist environments [16]. This contradiction suggests that environmental transitions, such as moving from an aquatic to a subaerial setting, may have been crucial for facilitating unexpected tissue desiccation. Although mummification is traditionally associated with arid environments, recent forensic research has shown that it can also occur under temperate or transitional climatic conditions when microenvironmental factors promote rapid desiccation [44, 45]. In the present study, the rapid transition from an aquatic to a subaerial environment likely created a unique moisture gradient that facilitated accelerated tissue drying, contributing to the observed mummification. This is a unique observation that has not been previously described and requires further investigation.

Further research may explore whether rapid drying due to variations in the combination of temperature and humidity changes influenced this outcome, resulting in the quick mummification observed on aquatic-to-subaerial group piglet bodies. Some researchers have reported that the warm and dry conditions of Cape Town’s coastal region facilitate the mummification of remains [46]. Similarly, the present study was conducted during a two-week period in 12/2024 (a summer month associated with relatively high temperatures and afternoon showers), when subaerial temperatures were relatively high and rainfall was minimal in Johannesburg. These conditions likely contributed to the rapid desiccation of aquatic-to-subaerial group piglet bodies following their removal from water, leading to the skin drying tightly around the skeletal frame. Furthermore, the concrete surface where the aquatic-to-subaerial remains were placed following the environmental transition may have influenced the observed decomposition patterns as studies have reported that piglet bodies positioned on concrete decompose at a slower rate and exhibit earlier onset of mummification compared to those placed on natural substrates such as grass [47, 48]. The delayed decomposition observed on concrete may be attributed to its antimicrobial properties, which stem from the material’s high alkalinity due to its composition [49]. Although external decomposition appeared to halt due to mummification, internal decomposition persisted, as evidenced by maggot activity and the presence of decomposition fluids leaking from orifices. The internal maggot activity would have reduced the water content and thus contributed to preserving the remains in a mummified state [50].

This study demonstrates that transitional decomposition can be experimentally modeled, providing a foundation for future studies to build designs that more closely reflect forensic realism. Subsequent research should incorporate larger sample sizes and longer PMIs to capture a broader range of morphological trajectories and late-stage changes. Future studies could also investigate whether submerged remains, when exposed to high temperatures and low humidity following retrieval from an aquatic environment, undergo a unique decomposition trajectory in which external mummification occurs while internal microbial and insect activity continues. Although this is a proof-of-concept study that only explores a controlled artificial body of water, it highlights an important limitation to the existing decomposition scoring systems: no validated aquatic decomposition scoring system exists exclusively for pig models, despite their widespread use as human analogues. Therefore, future studies should prioritize developing and calibrating a pig-specific aquatic scoring system rather than relying on adapted terrestrial tools or human-based models. In addition, expanding the research to include different water bodies, seasons, and sizes and coverings of remains would further refine the applicability of transitional decomposition findings to real-world forensic casework.

### Limitations and future studies

This study was designed as a proof-of-concept investigation to evaluate decomposition under controlled transitional environmental conditions. Although the findings provide novel insights into the effects of environmental transitions on decomposition, several limitations of the proof-of-concept study design should be acknowledged to direct the study designs of future research that can build upon the study results.

A limitation of this study is the use of juvenile piglets weighing between 1.5 and 6 kg. Previous research has demonstrated that carcass size can influence decomposition rates, insect colonization patterns, heat retention, and tissue persistence, with larger carcasses exhibiting different decomposition trajectories compared to smaller carcasses [51, 52]. Larger porcine models, often approximately 60 kg in mass, are generally considered more representative of adult human decomposition. Consequently, caution should be exercised when extrapolating the present findings to larger human remains. This study should be replicated with pigs with larger body mass to confirm if the findings are only limited to smaller body sizes.

Secondly, all piglet bodies died of natural causes prior to donation. Although natural deaths are not expected to substantially alter decomposition trajectories, the exact physiological state of each animal before death could not be standardized. Many decomposition studies utilize euthanized animals to ensure greater consistency in pre-death physiological conditions; however these also have their limitations and may impact the decomposition process [53, 54]. Future studies should investigate whether differences in cause of death influence decomposition patterns under transitional environmental conditions.

Thirdly, the aquatic environment consisted of treated freshwater containers rather than natural water bodies. Consequently, environmental variables such as water flow, depth variation, sediment interactions, aquatic scavenging activity, and fluctuating water chemistry were not represented. While this controlled approach reduced environmental variability and allowed the effects of environmental transitions to be isolated, it may not fully reflect decomposition processes occurring in natural aquatic settings.

An additional limitation relates to decomposition scoring systems. Although the ATBS and TADS were successfully applied in this study, neither system has been specifically validated for remains that transition between aquatic and subaerial environments. Furthermore, no validated aquatic decomposition scoring system currently exists for porcine models despite their widespread use as human analogues in forensic taphonomic research [17, 55]. The conversion of TADS values to an STBS also introduces the possibility of numerical bias when comparing decomposition progression across environments. This limitation highlights the need for the development and validation of decomposition scoring systems specifically designed for aquatic and transitional decomposition contexts and be appropriate for the study model (i.e. human or nonhuman animal models).

Future research should incorporate larger sample sizes, longer postmortem intervals, larger porcine models, multiple seasons, and a variety of aquatic environments to further evaluate the effects of environmental transitions on decomposition. Future studies should also investigate the mechanisms underlying the rapid mummification observed following aquatic-to-subaerial transitions and the accelerated tissue loss observed following subaerial-to-aquatic transitions. Ultimately, the development of validated decomposition scoring systems capable of accommodating environmental transitions may improve postmortem interval estimation and forensic interpretation in cases involving movement of remains between environments.

## Conclusions

The findings indicate that decomposition trajectories are strongly influenced by environmental transitions, resulting in outcomes that deviate from established terrestrial and aquatic patterns, which are more linear in pattern in stable environments. Piglet bodies transitioning from a subaerial to an aquatic environment exhibited accelerated decomposition following rehydration, culminating in rapid soft tissue loss and near-complete skeletonization. In contrast, piglet bodies transitioning from aquatic to subaerial conditions underwent rapid desiccation and mummification, which altered the apparent progression of decomposition and slowed further soft tissue breakdown.

The results suggest that decomposition scoring systems, particularly when adapted across environments, may not reliably capture these transitional processes. While STBS showed strong correlations with logADD in the subaerial-to-aquatic group, indicating potential applicability for PMI estimation, weaker correlations and reduced predictive reliability in the aquatic-to-subaerial group suggest limitations in current scoring methods following aquatic exposure. The use of different scoring frameworks (ATBS and TADS) further introduces inconsistencies that may affect the accurate assessment of decomposition of remains that transition between environments.

Morphological observations highlight the complexity of transitional decomposition, where rehydration enhances tissue breakdown and mummification preserves soft tissue, potentially obscuring true PMIs. These processes carry important forensic implications, particularly in cases where the environmental history of remains is unknown, as they may lead to misinterpretation of decomposition stage and PMI estimation.

These findings highlight the need for improved, standardized decomposition scoring systems that account for environmental transitions. Further research incorporating larger sample sizes, extended PMIs, and varied environmental conditions is required to better characterize these transitional decomposition pathways and improve the accuracy of forensic interpretations.

## Declarations

### Disclaimers

The authors conceived this study, and performed the data collection, analysis and interpretation, and the writing of this article. The manuscript, data, figures, and tables have not been previously published and are not under consideration elsewhere.
